# Effects of general and spinal anesthesia on postoperative rehabilitation in older adults after lower limb surgery: a retrospective cohort study

**DOI:** 10.3389/fmed.2024.1386797

**Published:** 2024-03-28

**Authors:** Guifei Li, Qingjing Ma, Yizhen Li, Furong Tan, Xuan Li, Jie Chen

**Affiliations:** ^1^Department of Anesthesiology, Shapingba District Hospital of Traditional Chinese Medicine, Chongqing, China; ^2^Department of Anesthesiology, The Second Affiliated Hospital of Chongqing Medical University, Chongqing, China

**Keywords:** general anesthesia, spinal anesthesia, Barthel score, older adults, lower limb surgery

## Abstract

**Objective:**

To investigate the effects of perioperative general anesthesia (GA) and spinal anesthesia (SA) on postoperative rehabilitation in elderly patients with lower limb surgery.

**Methods:**

This retrospective propensity score-matched cohort study included patients aged 65 years or older who underwent lower limb surgery between January 1, 2020, and May 31, 2023. The GA and SA were selected at the request of the orthopedic surgeon, patient, and their family members. The main outcomes included the incidence of the patient’s inability to self-care at discharge, postoperative complications including pulmonary infection, thrombus of lower extremity veins, infection of incisional wound and delirium, length of hospital stay, and incidence of severe pain in the first 2 days postoperatively.

**Results:**

In total, 697 patients met the inclusion criteria, and 456 were included in the final analysis after propensity score matching. In the GA and SA groups, 27 (11.84%) and 26 (11.40%) patients, respectively, could not care for themselves at discharge. The incidence rates did not differ between the groups (*p* = 0.884). In contrast, the incidence of postoperative complications (GA: 10.53% and SA: 4.39%; *p* = 0.013) and the length of hospital stay (GA: 16.92 ± 10.65 days and SA: 12.75 ± 9.15 days; *p* < 0.001) significantly differed between the groups.

**Conclusion:**

The choice of anesthesia is independent of the loss of postoperative self-care ability in older patients (>65 years) and is not a key factor affecting postoperative rehabilitation after lower limb surgery. However, compared with GA, SA reduces the incidence of postoperative complications and a prolonged hospital stay. Thus, SA as the primary anesthetic method is a protective factor against a prolonged hospital stay.

## 1 Introduction

The global population is aging, and older adults are affected by bone loss and physical aging, making them vulnerable to lower limb fractures. Consequently, the proportion of hip fractures is increasing, with an estimated 2.6 million cases worldwide by 2025 (1), most of which are caused by falling. The prognosis of lower extremity fractures in older patients is usually poor for many reasons, such as advanced age, sex, and underlying diseases (cardiovascular and cerebrovascular diseases, frailty, and cognitive dysfunction). Lower limb fractures in this patient population require surgery to restore mobility and relieve pain.

Effective and successful surgeries require anesthesia, which provides pain relief during the procedure to ensure the operation is smooth and safe. However, surgery also puts the patient at risk for various complications, including infection, thrombosis in the lungs or legs, bedsores, urinary tract infections, and pneumonia. For older or frail adults, rehabilitation may take months, depending on the patient’s age, comorbidities, and postoperative pain, as well as infection. The choice of anesthesia affects patients’ intraoperative hemodynamics and cardiopulmonary function, affecting their prognosis. Therefore, anesthesia has been controversial for older patients with lower limb fractures (2–8), but how anesthesia affects postoperative rehabilitation remains unknown.

Therefore, this study investigated the effects of general anesthesia (GA) and spinal anesthesia (SA) on postoperative rehabilitation in older adults undergoing lower extremity surgery, such as their postoperative self-care ability, postoperative complications, and hospital stay, aiming to provide a reference for selecting the optimal anesthesia method for these patients.

## 2 Methods

### 2.1 Study design

This was a retrospective propensity-score-matched cohort study. The Ethics Committee of the Shapingba District Hospital of Chongqing Municipality approved this study No: (2024) (03), which was conducted following the Declaration of Helsinki. The confidentiality of patient data was guaranteed owing to the study’s retrospective nature.

### 2.2 Patients

Patients who underwent lower limb surgery at the Shapingba District Hospital of Traditional Chinese Medicine in Chongqing between January 1, 2020, and May 31, 2023, were selected. The inclusion criteria were: (1) patients undergoing elective lower limb surgery (including total hip replacement, lower limb fracture, internal fixation, debridement, and other lower limb surgeries) under GA and SA and (2) patients older than 65 years. The exclusion criteria were: (1) receiving local anesthesia, nerve block or intravenous anesthesia, (2) postoperative death, and (3) lack of data on self-care assessment.

### 2.3 Observational indicators

Patient data were obtained from the hospital’s electronic record system and the SA follow-up system. The general data collected in this study included demographic information, such as sex, age, preoperative comorbidities, the American Society of Anesthesiologists classification, the preoperative and discharge Barthel scores, preoperative routine blood markers, liver and renal function, the visual analog scale (VAS) scores for postoperative pain after 2 days and at discharge, and the length of hospital stay. Comorbidities included cardiovascular diseases (coronary heart disease, hypertension, and arrhythmia), nervous system diseases (cerebral infarction and Parkinson’s disease), respiratory diseases (chronic obstructive pulmonary disease, pulmonary infection, and bronchitis), and other diseases. Other information related to surgery included the operation duration, intraoperative use of pressure pressor (yes/no), postoperative analgesia (intravenous analgesia, controlled analgesia/ward load dose analgesia mode), and nerve block use.

The Barthel score was used to assess self-care at discharge. In this study, a total score of 40 indicated severe self-care dysfunction (i.e., the inability to care for themselves). A total score of 41–60 indicated that auxiliary care was required, and a score of >60 indicated mild dysfunction, and only partial care was required (9–11). The main study index was the incidence of a Barthel score below 40 at discharge. The VAS was used to assess postoperative pain. Scores ≥5 were defined as severe pain, and patients with a VAS score of ≥5 at any period after surgery were classified with severe postoperative pain. A hospital stay longer than 2 weeks was defined as a prolonged hospital stay and a surgical duration of more than 2 h was defined as prolonged surgery.

Finally, patients were divided into GA and SA groups, and the incidences of patients unable to take care of themselves at discharge, postoperative complications, and prolonged hospitalization were analyzed.

### 2.4 Sample size calculation

Statistical software PASS version 11.0 (Copyright 2011 NCSS, LLC) was used for the Sample size calculation. The main outcome of this study was the incidence of postoperative self-care. According to the results of previous literature (12), GA (48%) and SA (31%), a minimum of 258 evaluable patients (129 per arm) would be required to detect a difference between the two groups at *p* = 0.05 (2-tailed), with a power of 90%. Thus, the sample size is enough for the current study.

### 2.5 Statistical analyses

Statistical software SPSS version 26.0 (IBM Corp., Armonk, NY, United States) was used for the data analyses. Normally distributed measurement data were expressed as X ± S, and qualitative variables as the number of cases and percentages. Normally distributed continuous data were evaluated using independent sample *t*-tests, non-normally distributed measurement data were analyzed with the Mann–Whitney *U* test and expressed as M (*P*_25_, *P*_75_), and qualitative data were analyzed with X for qualitative data^2^ checkout. *p*-values of <0.05 indicated statistical significance.

To address possible confounders, propensity-score matching (PSM) was used to validate the study rationale and methods. To investigate the predictors of postoperative rehabilitation, univariate and multivariate logistic regression analyses were performed for postoperative self-care ability, postoperative complications, and prolonged hospital stay. Variables with *p*-values <0.05 in the univariate analysis were used in the multivariate analysis. In addition to anesthesia, the model included age, sex, comorbidities, length of hospitalization, postoperative analgesia, combined nerve block, and routine preoperative blood test indicators.

## 3 Results

**Figure 1:**
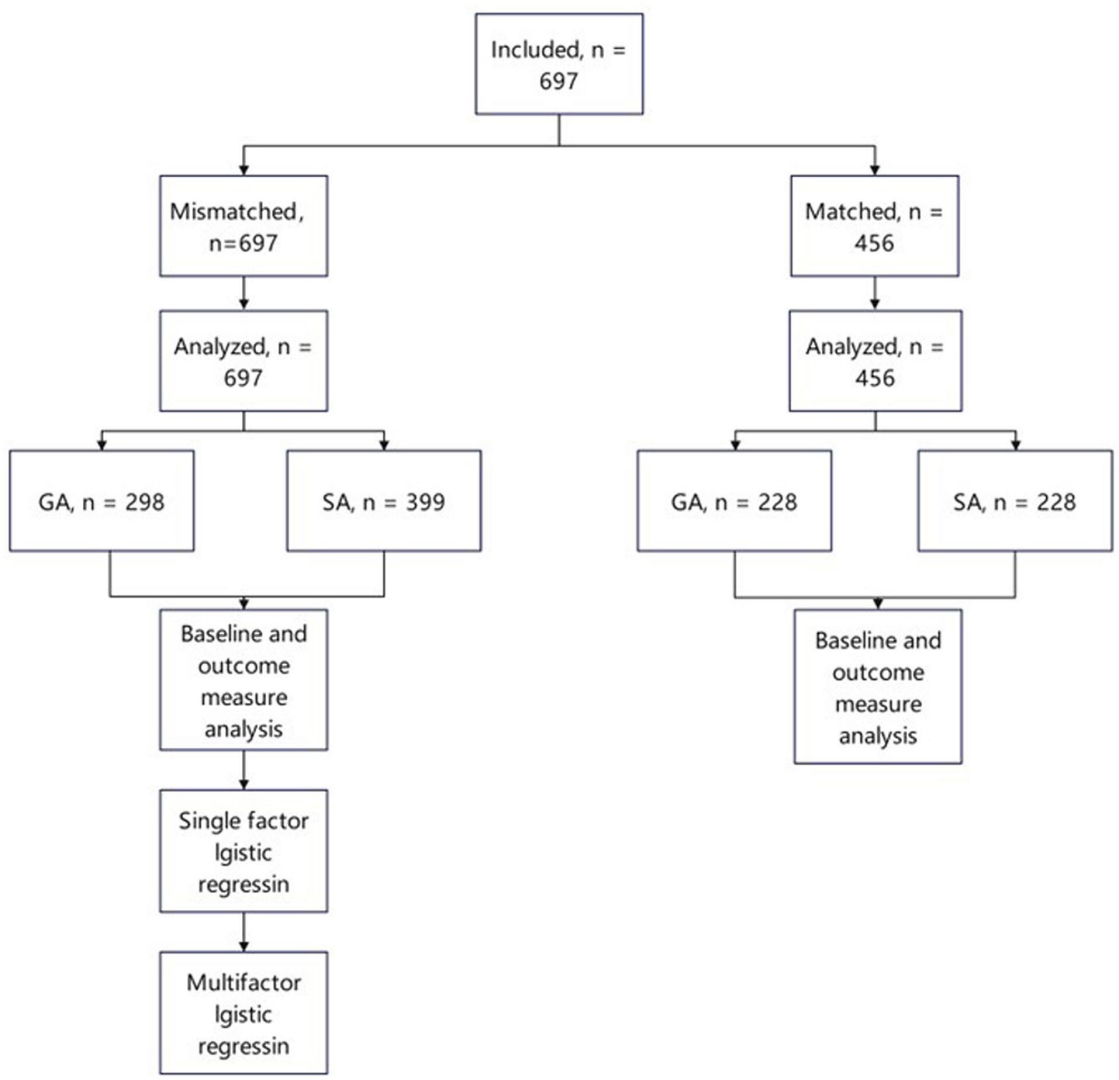
Participant flow chart. GA, general anesthesia; SA, spinal anesthesia.

Figure 1 presents a flowchart of the participant selection process. In total, 697 patients met the inclusion criteria. Before PSM, the baseline data for age (*p* < 0.001), sex (*p* = 0.001), and combined nerve block (*p* < 0.001) were unbalanced between the groups. Therefore, PSM (caliper value = 0.02) was used to reduce selection bias and potential baseline differences between the GA and SA groups. After PSM, the distributions of the background variables were well-balanced, and 456 patients were included in the analysis. Tables 1, 2 detail the baseline comparisons before and after matching, respectively.

**Table 1:** Baseline characteristics of all patients.

	General anesthesia (*n* = 298)	Spinal anesthesia (*n* = 399)	Difference value and 95% confidence interval (CI)	*p*-value
Age (X ± S, year)	72.56 ± 6.76	77.16 ± 8.58	4.56 (5.78–3.42)	0.000
Sex [*n* (%)]			11.61 (18.11–4.99)	0.001
Male	61 (20.47)	128 (32.08)		
Female	237 (79.53)	271 (67.92)		
ASA classification [*n* (%)]			11.36 (4.89–17.83)	0.001
II	237 (79.53)	272 (68.17)		
III	61 (20.47)	127 (31.83)		
Comorbidities [*n* (%)]			2.78 (4.67–10.23)	0.464
Yes	162 (54.36)	228 (57.14)		
No	136 (45.64)	171 (42.86)		
Preoperative Barthel [*n* (%)]			5.28 (0.48–11.04)	0.077
41–100	251 (84.23)	315 (78.95)		
≤40	47 (15.77)	84 (21.05)		
Nerve block			10.07 (4.91–15.23)	0.000
Yes	269 (90.27)	320 (80.20)		
No	29 (9.73)	79 (19.80)		
WBC count <9.5 × 10^9^/L [*n* (%)]	38 (12.75)	66 (16.54)	2.11 (3.51–6.81)	0.176
PLT count <25 × 10^9^/L [*n* (%)]	43 (14.43)	51 (12.78)	1.65 (3.51–6.81)	0.517
HB <115 g/L [*n* (%)]	87 (29.19)	149 (37.34)	8.15 (1.14–15.16)	0.026
Albumin <40 g/L [*n* (%)]	123 (41.28)	216 (54.14)	12.86 (5.44–20.28)	0.001
ALT >40 U/L [*n* (%)]	15 (5.03)	17 (4.26)	0.77 (2.40–3.94)	0.621
AST >45 U/L [*n* (%)]	11 (3.69)	9 (2.26)	1.43 (1.16–4.02)	0.260
Creatinine >80 μmol/L [*n* (%)]	53 (17.79)	105 (26.32)	8.53 (2.41–14.65)	0.009

ASA, American Society of Anesthesiologists; WBC, white blood cell; PLT, blood platelet; HB, hemoglobin; ALT, alanine aminotransferase; AST, aspartate aminotransferase.

**Table 2:** Baseline characteristics in propensity score-matched cohorts.

	General anesthesia (*n* = 228)	Spinal anesthesia (*n* = 228)	Difference value and 95% CI	*p*-value
Age (X ± S, year)	73.57 ± 7.21	73.81 ± 8.11	0.23 (1.65–1.18)	0.058
Sex [*n* (%)]			0 (0–0)	1.000
Male	60 (26.3)	60 (26.3)		
Female	168 (73.7)	168 (73.7)		
ASA classification [*n* (%)]			0.5 (7.28–8.28)	0.912
II	175 (76.8)	174 (76.3)		
III	53 (23.2)	54 (23.7)		
Comorbidities [*n* (%)]			6.14 (2.98–15.26)	0.188
Yes	132 (57.89)	118 (51.75)		
No	96 (42.11)	110 (48.25)		
Preoperative Barthel [*n* (%)]			1.76 (5.01–8.53)	0.611
41–100	189 (82.89)	193 (84.65)		
≤40	39 (17.11)	35 (15.35)		
Nerve block			5.71 (0.95–12.37)	0.093
Yes	199 (87.28)	186 (81.57)		
No	29 (12.92)	42 (18.42)		
WBC count <9.5 × 10^9^/L [*n* (%)]	24 (10.53)	33 (14.47)	3.94 (2.12–10.00)	0.202
PLT count <125 × 10^9^/L [*n* (%)]	37 (16.23)	28 (12.28)	3.95 (2.46–10.36)	0.221
HB <115 g/L [*n* (%)]	70 (30.70)	63 (27.63)	3.07 (5.27–11.41)	0.452
Albumin <40 g/L [*n* (%)]	94 (41.23)	89 (39.04)	2.19 (6.81–11.19)	0.578
ALT >40 U/L [*n* (%)]	8 (3.50)	9 (3.95)	0.45 (3.03–3.93)	0.805
AST >45 U/L [*n* (%)]	7 (3.07)	6 (2.63)	0.44 (2.61–3.49)	0.778
Creatinine >80 μmol/L [*n* (%)]	44 (19.30)	47 (20.61)	1.31 (6.03–8.65)	0.761

CI, confidence interval; ASA, American Society of Anesthesiologists; WBC, white blood cell; PLT, blood platelet; HB, hemoglobin; ALT, alanine aminotransferase; AST, aspartate aminotransferase.

After PSM, 27 (11.84%) and 26 (11.40%) patients in the GA and SA groups, respectively, were unable to care for themselves at discharge; the incidence rate did not differ between the groups (*p* = 0.884). In contrast, the incidence of postoperative complications (e.g., pulmonary infection, lower limb venous thrombosis, incision infection, and postoperative delusion) (GA: 10.53% and SA: 4.39%; *p* = 0.013) and length of hospital stay (GA: 16.92 ± 10.65 days and SA: 12.75 ± 9.15 days; *p* < 0.001) significantly differed between the groups. The VAS scores 2 days postoperatively and on the day of discharge did not differ between the groups (*p* > 0.05). Thus, the anesthesia method was independent of the incidence of self-care dysfunction at discharge, prolonged hospital stay, and the rate of postoperative complications. The use of intravenously controlled analgesia varied greatly after surgery; however, there was an incidence of severe pain on the first day and the day of discharge (Tables 3, 4).

**Table 3:** Postoperative observation indicators before propensity score matching.

	General anesthesia (*n* = 298)	Spinal anesthesia (*n* = 399)	*p*-value
Barthel at discharge [*n* (%)]			0.043
≤40	30 (10.07)	61 (15.29)	
>40	268 (89.93)	338 (84.71)	
Postoperative complications [*n* (%)]	24 (8.05)	23 (5.76)	0.304
Pulmonary infection	12	15	0.856
Thrombus of lower extremity veins	5	3	0.438
Infection of incisional wound	5	4	0.658
Delirium	2	1	0.799
LOS (X ± S, day)	16.59 ± 9.97	13.51 ± 8.64	0.476
Prolonged LOS [*n* (%)]	156 (52.35)	145 (36.34)	0.000
Severe pain on the first day after surgery [*n* (%)]	134 (44.97)	151 (37.84)	0.084
Severe pain on the second day after surgery [*n* (%)]	62 (20.81)	82 (20.55)	0.967

LOS, length of stay.

**Table 4:** Postoperative observation indicators after propensity score matching.

	General anesthesia (*n* = 228)	Spinal anesthesia (*n* = 228)	*p*-value
Barthel at discharge [*n* (%)]			0.884
≤40	27 (11.84)	26 (11.40)	
>40	201 (88.16)	202 (88.60)	
Postoperative complications [*n* (%)]	24 (10.53)	10 (4.39)	0.013
Pulmonary infection	12	6	0.149
Thrombus of lower extremity veins	5	0	0.025
Infection of incisional wound	5	3	0.721
Delirium	2	1	0.562
LOS (X ± S, day)	16.92 ± 10.65	12.75 ± 9.15	0.000
Prolonged LOS [*n* (%)]	123 (53.95)	74 (32.46)	0.000
Severe pain on the first day after surgery [*n* (%)]	102 (44.73)	90 (39.47)	0.225
Severe pain on the second day after surgery [*n* (%)]	45 (19.74)	51 (22.37)	0.491

LOS, length of stay.

**Figure 2:**
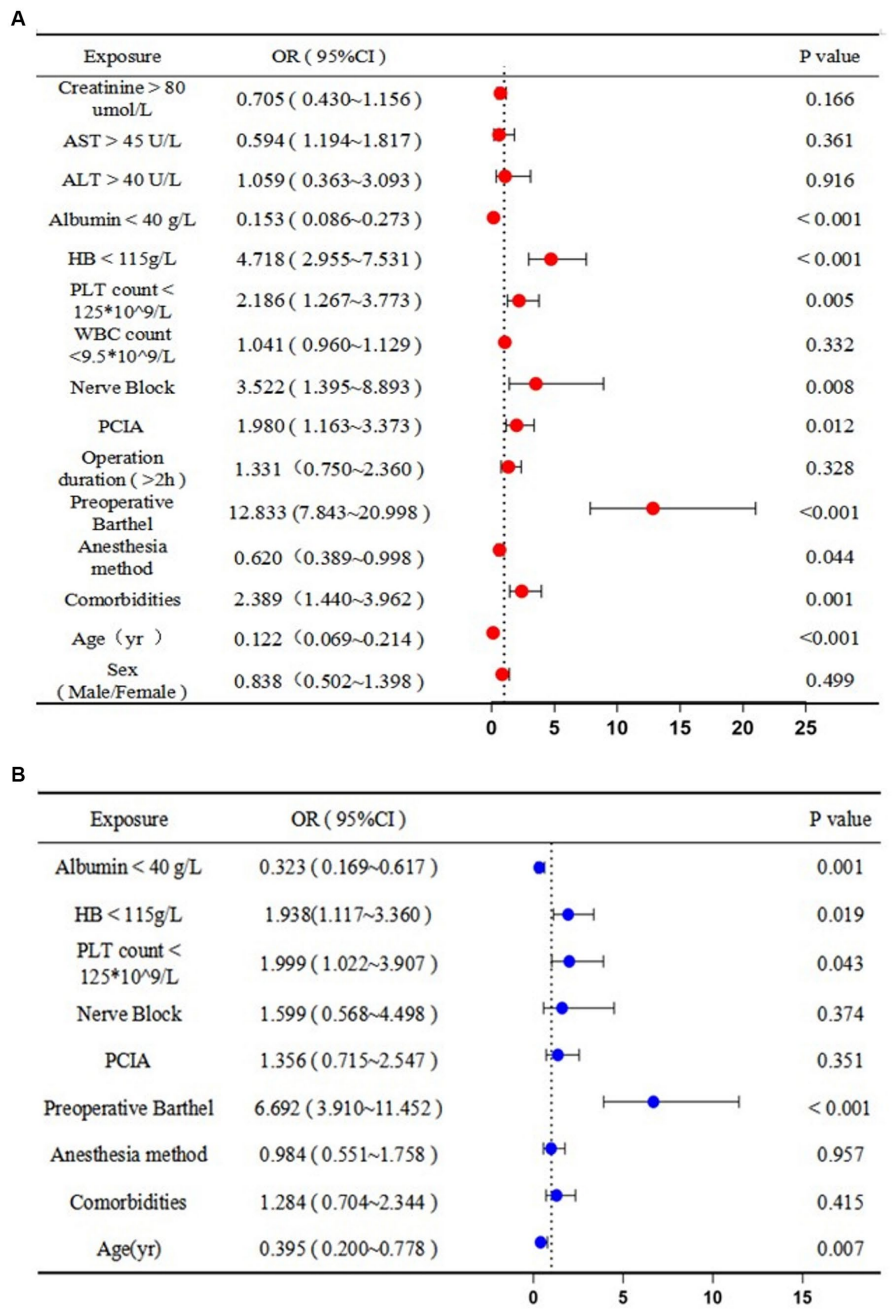
Predictors of postoperative disability (i.e., inability to self-care): **(A)** univariate and **(B)** multivariate logistic regression analyses. OR, odds ratio; CI, confidence interval; AST, aspartate aminotransferase; ALT, alanine aminotransferase; HB, hemoglobin; PLT, platelet; WBC, white blood cell; PCIA, patient-controlled intravenous analgesia.

The univariate analysis demonstrated that postoperative self-care ability was associated with increased age [0.122 years, 95% confidence interval (CI): 0.069 to 0.214, *p* < 0.05], comorbidities (2.389, 95% CI: 1.440 to 3.962, *p* < 0.05), anesthesia method (0.620, 95% CI: 0.389 to 0.998, *p* < 0.05), preoperative Barthel score (12.833, 95% CI: 7.843 to 20.998, *p* < 0.001), patient-controlled intravenous analgesia (PCIA; 1.980, 95% CI: 1.163 to 3.373, *p* < 0.05), nerve block (3.552, 95% CI: 1.395 to 8.893, *p* < 0.05), decreased platelet (PLT) count (2.186 × 10^9^/L, 95% CI: 1.267 to 3.773, *p* < 0.05), decreased hemoglobin (HB) levels (4.718 g/L, 95% CI: 2.955 to 7.531, *p* < 0.001), and decreased albumin levels (0.153 g/L, 95% CI: 0.086 to 0.273, *p* < 0.001). Increased age (0.395 years, 95% CI: 0.200 to 0.778, *p* < 0.05), preoperative Barthel score (6.692, 95% CI: 3.910 to 11.542, *p* < 0.001), decreased PLT count (1.999 × 10^9^/L, 95% CI: 1.022 to 3.907, *p* < 0.05), decreased HB levels (1.938 g/L, 95% CI: 1.117 to 3.360, *p* < 0.05), and decreased albumin levels (0.323 g/L, 95% CI: 0.169 to 0.617, *p* < 0.05) were associated with postoperative self-care ability in the multivariate analysis. Therefore, age and normal albumin levels were protective factors, and the inability to self-care preoperatively, a low PLT count, and anemia were risk factors for the inability to self-care postoperatively (Figure 2).

**Figure 3:**
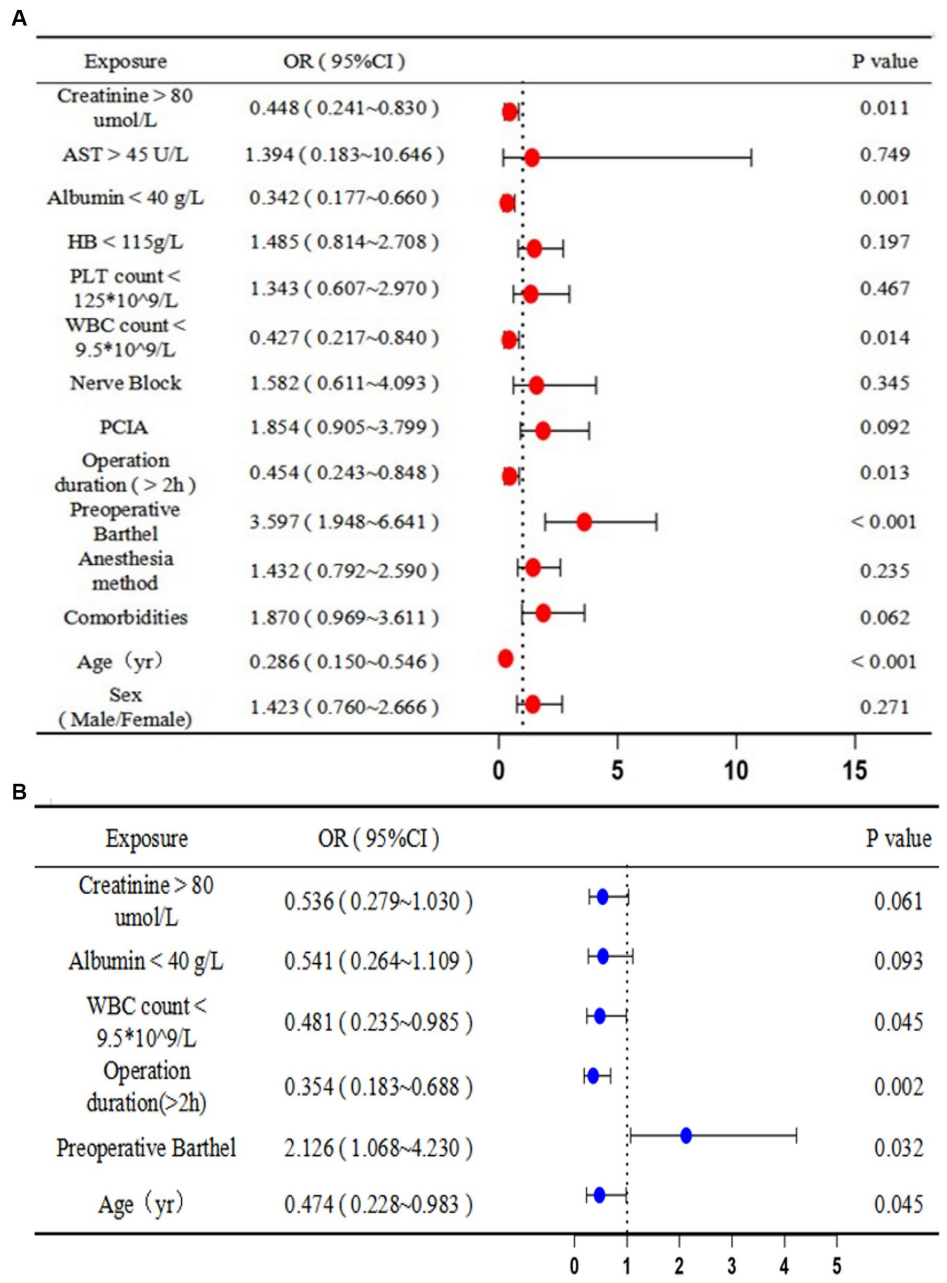
Predictors of postoperative complications: **(A)** univariate and **(B)** multivariate logistic regression analyses. OR, odds ratio; CI, confidence interval; AST, aspartate aminotransferase; ALT, alanine aminotransferase; HB, hemoglobin; PLT, platelet; WBC, white blood cell; PCIA, patient-controlled intravenous analgesia.

The univariate analysis demonstrated that postoperative complications were associated with increased age (0.286 years, 95% CI: 0.150 to 0.546, *p* < 0.001), preoperative Barthel score (3.597, 95% CI: 1.948 to 6.641, *p* < 0.001), operation duration >2 h (0.454 h, 95% CI: 0.243 to 0.848, *p* < 0.05), increased white blood cell (WBC) count (0.427 × 10^9^/L, 95% CI: 0.217 to 0.840, *p* < 0.05), decreased albumin levels (0.342 g/L, 95% CI: 0.177 to 0.660, *p* < 0.05), and increased creatinine levels (0.448 μmol/L, 95% CI: 0.241 to 0.830, *p* < 0.05). Increased age (0.474 years, 95% CI: 0.228–0.983, *p* < 0.05), preoperative Barthel score (2.126, 95% CI: 1.068–4.230, *p* < 0.05), operation duration >2 h (0.354 h, 95% CI: 0.183–0.688, *p* < 0.05), and WBC count (0.481 × 10^9^/L, 95% CI: 0.235–0.985, *p* < 0.05) were associated with postoperative complications in the multivariate analysis. Therefore, younger age, an operative time of less than 2 h, and a normal WBC count were protective factors, and the inability to self-care preoperatively was a risk factor for postoperative complications (Figure 3).

**Figure 4:**
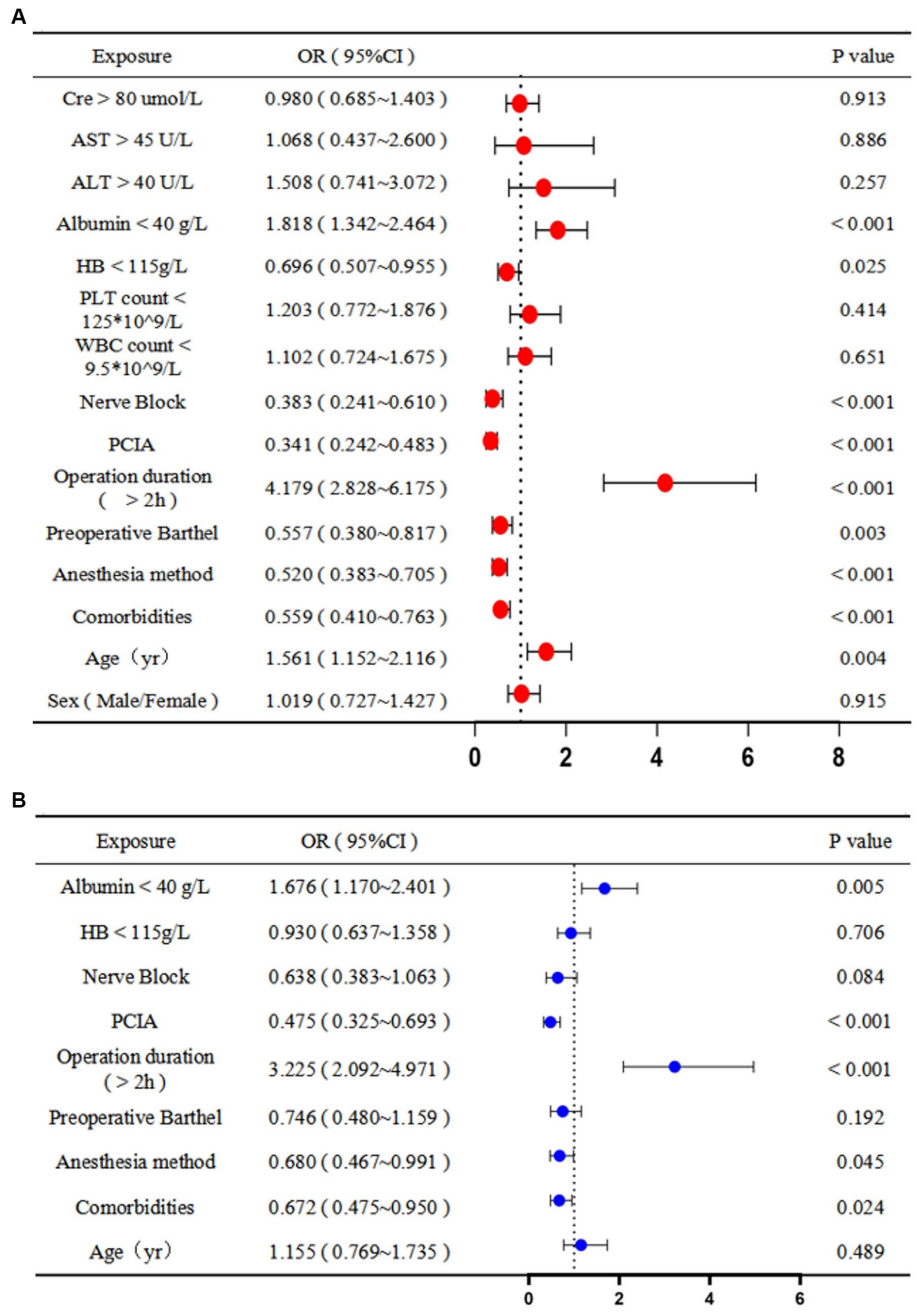
Predictors of prolonged hospital stay: **(A)** univariate and **(B)** multivariate logistic regression analyses. OR, odds ratio; CI, confidence interval; AST, aspartate aminotransferase; ALT, alanine aminotransferase; HB, hemoglobin; PLT, platelet; WBC, white blood cell; PCIA, patient-controlled intravenous analgesia.

The univariate analysis demonstrated that prolonged hospital stay was associated with increased age (1.561 years, 95% CI: 1.152 to 2.116, *p* < 0.05), comorbidities (0.559, 95% CI: 0.410 to 0.763, *p* < 0.001), anesthesia method (0.520, 95% CI: 0.383 to 0.705, p < 0.001), preoperative Barthel score (0.557, 95% CI: 0.380 to 0.817, *p* < 0.05), operation duration >2 h (4.179 h, 95% CI: 2.828 to 6.175, *p* < 0.001), PCIA (0.341, 95% CI: 0.242 to 0.483, *p* < 0.001), nerve block (0.383, 95% CI: 0.241 to 0.610, *p* < 0.001), decreased HB level (0.696 g/L, 95% CI: 0.507 to 0.955, *p* < 0.05), and decreased albumin level (1.818 g/L, 95% CI: 1.342 to 2.464, *p* < 0.001). Comorbidities (0.672, 95% CI: 0.475 to 0.950, *p* < 0.05), anesthesia method (0.680, 95% CI: 0.467 to 0.991, *p* < 0.05), operation duration >2 h (3.225 h, 95% CI: 2.092 to 4.971, *p* < 0.001), PCIA (0.475, 95% CI: 0.325 to 0.693, *p* < 0.001), and decreased albumin levels (1.676 g/L, 95% CI: 1.170 to 2.401, *p* < 0.05) were associated with prolonged hospital stay in the multivariate analysis. Therefore, the absence of comorbidities, anesthetic method, and postoperative use of PCIA were protective factors, and a surgery duration longer than 2 h and low preoperative albumin levels were risk factors for a prolonged hospital stay (Figure 4).

## 4 Discussion

As society advances, the proportion of adults over the age of 65 years in China is increasing. According to a survey, there are currently approximately 280 million older adults in China, accounting for approximately 17.5% of the total population (13). However, physical and organ function gradually declines with age, and older individuals often develop various diseases, negatively affecting postoperative recovery. The choice of anesthesia can also affect recovery in older patients; therefore, selecting the appropriate method for this patient population is crucial for promoting recovery and reducing pain.

GA and SA are commonly used for lower limb fractures, and their effects on patients after hip fracture and total knee replacement surgeries have been investigated (14–17). However, the sample sizes of those studies were small, and no studies have explored how GA and SA affect self-care ability and length of stay in older patients. We found that the anesthesia method did not contribute to the loss of postoperative self-care ability in patients over age 65 years. However, we found that SA reduced the incidence of postoperative complications compared to GA and that SA was a protective factor against a prolonged hospital stay. And reducing postoperative complications and hospitalization time are important indicators for accelerating postoperative rehabilitation. There are studies have reported that SA may be associated with a reduced incidence of postoperative complications, including death, deep vein thrombosis, pulmonary complications, and myocardial infarction (2, 3, 12, 15, 18), which aligns with our results.

Selection bias is an obvious limitation of observational studies, resulting in unreliable results if improperly addressed. Logistic regression and PSM are two common statistical methods for reducing selection bias. PSM is a data processing method used to control for confounding factors in observational and retrospective studies and is often used in orthopedic studies (19). This study included 697 patients based on the inclusion and exclusion criteria. However, certain characteristics (e.g., age, sex, and nerve block use) significantly differed between the two groups prior to PSM. However, after PSM, 456 patients were included, and significant differences in the general or preoperative data were not observed between the two groups. Subsequently, logistic regression was used to analyze and predict the factors affecting postoperative rehabilitation.

This study has some limitations. First, only patients who underwent lower limb surgery were included, and the anesthesia method was mainly GA with a spinal nerve block; the type of nerve block medication and nerve block needle nor the type or dose of systemic anesthesia or SA were assessed. Therefore, the applicability of these results for patients receiving other types of anesthesia remains to be clarified. Second, we did not collect data on several variables that may affect the results, such as body mass index, although a high body mass index has been associated with surgical site infection after total hip replacement (20). We also did not collect postoperative routine blood test data despite an independent association between a neutrophil-to-lymphocyte ratio of >10.5 and severe pain within 48 h postoperatively (21).

Although this study cannot definitively support one mode of anesthesia over another, the results highlight each method’s relative risks and benefits, especially SA, which was a protective factor against a prolonged length of stay. Overall, these results provide objective evidence for selecting an anesthesia method for lower limb surgery to encourage rapid postoperative recovery.

## Data availability statement

The raw data supporting the conclusions of this article will be made available by the authors, without undue reservation.

## Ethics statement

The studies involving humans were approved by the Ethics Committee of the Shapingba District Hospital of Chongqing Municipality. The studies were conducted in accordance with the local legislation and institutional requirements. The ethics committee/institutional review board waived the requirement of written informed consent for participation from the participants or the participants’ legal guardians/next of kin because this was a retrospective propensity-score-matched cohort study. The manuscript presents research on animals that do not require ethical approval for their study.

## Author contributions

GL: Data curation, Investigation, Validation, Writing – original draft. QM: Data curation, Formal analysis, Writing – original draft. YL: Data curation, Writing – original draft. FT: Data curation, Writing – original draft. XL: Data curation, Investigation, Writing – original draft. JC: Supervision, Validation, Writing – original draft, Writing – review & editing.
